# Diagnostic Utility of SOX4 Expression in Adult T-Cell Leukemia/Lymphoma

**DOI:** 10.3390/diagnostics11050766

**Published:** 2021-04-24

**Authors:** Atsuko Nasu, Yuka Gion, Yoshito Nishimura, Asami Nishikori, Misa Sakamoto, Yuria Egusa, Azusa Fujita, Tadashi Yoshino, Yasuharu Sato

**Affiliations:** 1 Division of Pathophysiology, Okayama University Graduate School of Health Sciences, Okayama 700-8558, Japan; nasu-a@cc.okayama-u.ac.jp (A.N.); asami.kei@s.okayama-u.ac.jp (A.N.); pls84dfj@s.okayama-u.ac.jp (M.S.); pb4k7y8z@s.okayama-u.ac.jp (Y.E.); ptqe7laf@s.okayama-u.ac.jp (A.F.); 2 Division of Anatomic Pathology, Okayama University Hospital, Okayama 700-8558, Japan; 3 Department of General Medicine, Okayama University Hospital, Okayama 700-8558, Japan; nishimura-yoshito@okayama-u.ac.jp; 4 Department of Medicine, John A. Burns School of Medicine, University of Hawai’i, Honolulu, HI 96813, USA; 5 Department of Pathology, Dentistry and Pharmaceutical Sciences, Okayama University Graduate School of Medicine, Okayama 700-8558, Japan; yoshino@md.okayama-u.ac.jp

**Keywords:** SOX4, p16, adult T-cell leukemia/lymphoma, peripheral T-cell lymphoma, not otherwise specified

## Abstract

Differentiation between adult T-cell leukemia/lymphoma (ATLL) and peripheral T-cell lymphoma, not otherwise specified (PTCL-NOS), is often challenging based on pathological findings alone. Although serum anti-HTLV-1 antibody positivity is required for ATLL diagnosis, this information is often not available at the time of pathological diagnosis. Therefore, we examined whether the expression of SOX4 and p16 would be helpful for differentiating the two disease entities. We immunohistochemically examined SOX4 and p16 expression (which have been implicated in ATLL carcinogenesis) in 11 ATLL patients and 20 PTCL-NOS patients and classified them into four stages according to the percentage of positive cells. Among the ATLL cases, 8/11 (73%) were SOX4-positive, while only 2/20 (10%) PTCL-NOS cases expressed SOX4. The mean total score was 4.2 (standard deviation (SD): 0.61) in the ATLL group and 0.50 (SD: 0.46) in the PTCL-NOS group (*p* < 0.001). Positive expression of p16 was noted in 4/11 (36%) patients with ATLL and 3/20 (15%) patients with PTCL-NOS, with mean total scores of 1.9 (SD: 0.64) and 0.70 (SD: 0.48) in the ATLL and PTCL-NOS groups, respectively (*p* = 0.141). These results suggest that SOX4 may be strongly expressed in ATLL compared to PTCL-NOS cases. Therefore, it may be helpful to perform immunohistochemical staining of SOX4 when pathologists face challenges discriminating between ATLL and PTCL-NOS.

## 1. Introduction

Adult T-cell leukemia/lymphoma (ATLL) is an aggressive mature T-cell neoplasm that is typically composed of highly pleomorphic lymphoid cells [1]. ATLL is caused by infection with human T-cell leukemia virus type-1 (HTLV-1) and it is a relatively common disease in southwestern Japan, Central Africa, and Latin America [1,2,3]. While patients with HTLV-1 do not necessarily develop ATLL, the distribution of the disease is closely linked to the prevalence of HTLV-1 in the population. ATLL cells, which are known as flower cells based on their morphological features in peripheral blood smears [4,5,6], proliferate both in peripheral blood and lymph nodes, and lymphadenopathy is often noted in patients with ATLL. Although the results of tests for serum anti-HTLV-1 antibodies are important and required for the diagnosis of ATLL, the results are not always available at the time of pathological diagnosis. Therefore, distinguishing between ATLL and other T-cell lymphomas is sometimes difficult. Peripheral T-cell lymphoma, not otherwise specified (PTCL-NOS), is a category of heterogeneous nodal and extra-nodal mature T-cell lymphomas that do not correspond to any specifically defined entities of mature T-cell lymphoma in the current classification system [7]. PTCL-NOS is an aggressive lymphoma requiring combination chemotherapy, such as cyclophosphamide, doxorubicin, vincristine, etoposide, and prednisone (CHOEP), which was shown to be an improvement on the currently used treatment regimen in a recent clinical trial [8]. In contrast, the VCAP (vincristine, cyclophosphamide, doxorubicin, and prednisone)-AMP (doxorubicin, ranimustine, and prednisone)-VECP (vindesine, etoposide, carboplatin, and prednisone) regimen is recommended as the first choice for patients with ATLL [9,10]. Due to the differences in treatment and prognosis, it is crucial to discriminate between these two diseases. However, pathological differentiation between ATLL and PTCL-NOS is often challenging using morphology alone. Recently, a transcription factor gene, SRY-box transcription factor 4 (*SOX4*), and the cyclin-dependent kinase inhibitor 2A (*CDKN2A*), have attracted the attention of researchers as they may play essential roles in lymphoma oncogenesis [11,12,13]. The human *CDKN2A* gene encodes a tumor suppressor protein known as p16. This cyclin-dependent kinase inhibitor regulates the G1 phase of the cell cycle and it is frequently inactivated in cancer [14]. In contrast, SOX4 is frequently overexpressed in a variety of solid tumors, where it functions as an oncogene [11]. SOX4 is a member of the SOX (Sry-related high-mobility group box) family of transcription factors that share homology in their DNA-binding domain, the high mobility group box. SOX4 regulates transcription through numerous methods, mediating both gene activation and repression [15]. This transcriptional regulator is known to form a complex with other proteins and plays a functional role in the apoptosis pathway, leading to cell death and tumor formation. SOX4 protein is overexpressed in various malignancies such as lung, breast, and prostate cancer, and it is closely associated with cancer migration and invasion [16,17,18,19]. Importantly, SOX4 plays crucial roles in embryonic development [19], including the developmental processes that give rise to T-cells and B-cells [11].

In this study, we examined whether immunohistochemical determination of SOX4 and p16 expression may be useful for pathological differentiation of ATLL and PTCL-NOS.

## 2. Materials and Methods

### 2.1. Samples

We analyzed the clinicopathological features of 31 patients who had been diagnosed with ATLL (11 patients) and PTCL-NOS (20 patients), and whose records were selected from pathology files in the Department of Pathology at Okayama University (Okayama, Japan). This study was approved by the Institutional Review Board of Okayama University (reference number 1607-016) and comprehensive informed consent was obtained for all subjects in the form of opt-out.

### 2.2. Immunohistochemical Staining for SOX4 and p16

Specimens were fixed in 10% formaldehyde and embedded in paraffin. Three-micrometer-thick sections were cut from the paraffin-embedded tissue blocks and stained with hematoxylin and eosin. Paraffin sections of each tissue sample were used for immunohistochemical staining with antibodies to SOX4 (polyclonal antibody, ab86809, dilution 1:60; Abcam, Cambridge, MA, USA) and p16 (clone: JC8, dilution 1:1000; Santa Cruz Biotechnology, Dallas, TX, USA). Immunohistochemical staining was performed using the automated Bond Max Stainer (Leica Biosystems, Wetzlar, Germany).

### 2.3. Evaluation of Immunohistochemical Staining for SOX4 and p16

**Figure 1:**
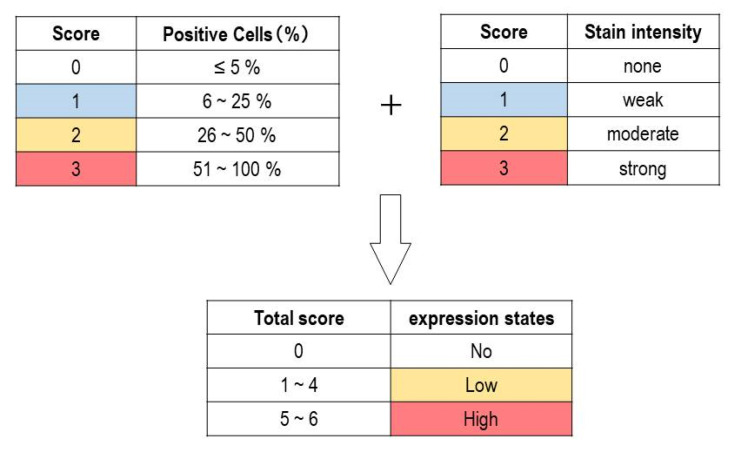
Immunohistochemical assessment of p16 in adult T-cell leukemia/lymphoma (ATLL) and peripheral T-cell lymphoma, not otherwise specified (PTCL-NOS). Evaluation criteria for staining score. The total score was calculated by adding the staining intensity score (0–3) and positive cell percentage score (0–3). The total score was stratified into three groups: no expression (score 0), low expression (score 1–4), and high expression (score 5–6).

After immunohistochemical staining for SOX4 and p16, specimens were assigned to one of four categories based on the percentage of tumor cells that were positive for SOX4 or p16. We defined the categories as follows: 0–5% positive cells (score of 0), 6–25% (1), 26–50% (2), and 51–100% (3). The evaluation was performed independently by one board-certified pathologist and two medical laboratory scientists. Next, the results of immunohistochemical staining were evaluated by expression intensity and range of positive cells. The intensity score was assessed on a scale of 0 to 3+: negative (0), weak positive (1+), moderate positive (2+), and strong positive (3+). Then, the total score was calculated by adding the intensity (0–3) and the range (0–3) scores. The total score was stratified into three groups: no expression (score 0), low expression (score 1–4), and high expression (score 5–6) (Figure 1). Our research team, including two board-certified pathologists, developed the scoring system.

### 2.4. Statistical Analysis

We analyzed the data using JMP version 15.1.0 (SAS Institute Inc., Cary, NC, USA). We used the Wilcoxon Rank Sum test to examine the differences between the two groups. The threshold for significance was defined as *p* < 0.05.

## 3. Results

### 3.1. Histological Features

**Figure 2:**
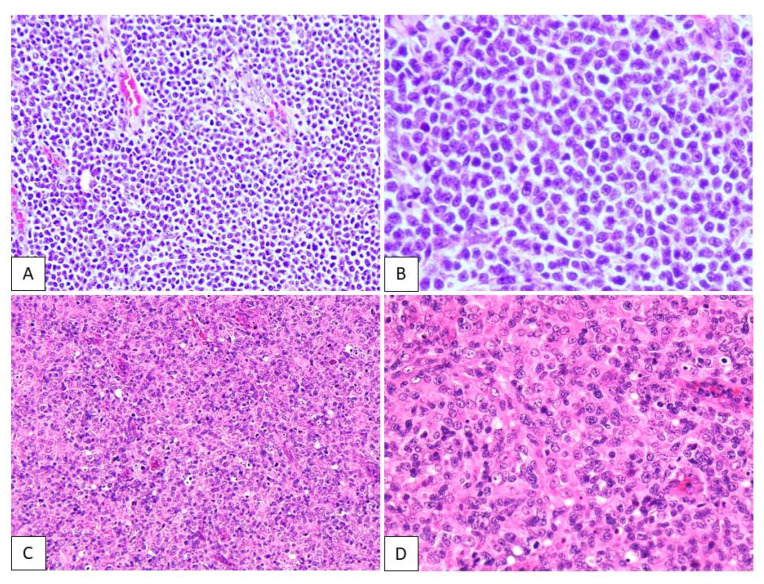
Hematoxylin and eosin staining in adult T-cell leukemia/lymphoma (ATLL) and peripheral T-cell lymphoma, not otherwise specified (PTCL-NOS). The neoplastic lymphoid cells in ATLL are typically medium to large, and the nuclear chromatin is coarsely clumped with distinct (**A**,**B**). The neoplastic lymphoid cells in PTCL-NOS are numerous medium-sized and/or large cells with irregular, pleomorphic, hyperchromatic, or prominent nuclei (**C**,**D**).

Representative figures showing lymphoid cells from patients with ATLL or PTCL-NOS are shown in Figure 2. The neoplastic lymphoid cells in the ATLL samples were typically medium to large in size, and the nuclear chromatin was coarsely clumped with distinct (Figure 2A,B). The neoplastic lymphoid cells in PTCL-NOS specimens comprised numerous medium and/or large cells with irregular, pleomorphic, hyperchromatic, or prominent nuclei (Figure 2C,D).

### 3.2. SOX4-Postive Scoring of ATLL and PTCL-NOS Specimens

**Figure 3:**
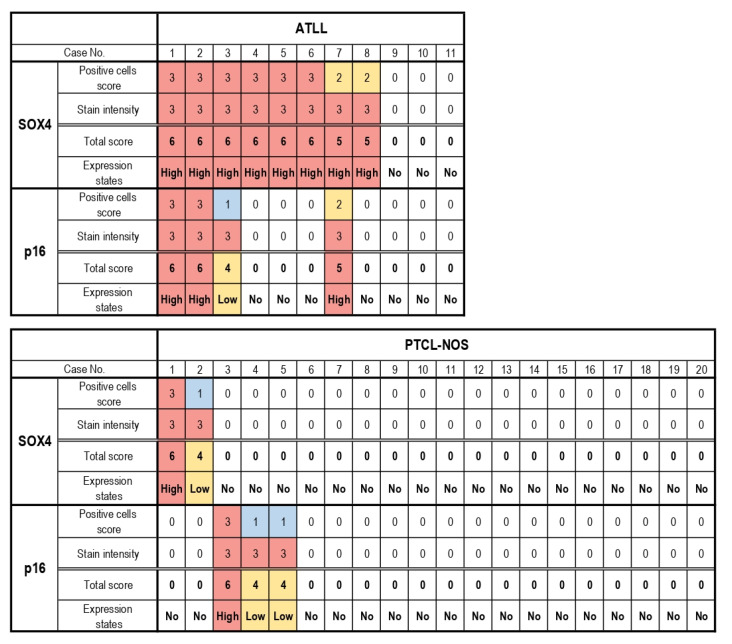
Heatmap representation of SOX4 and p16 immunostaining scores. A total of 8 cases with adult T-cell leukemia/lymphoma (ATLL) and 1 case with peripheral T-cell lymphoma, not otherwise specified (PTCL-NOS) exhibited high SOX4 expression. High p16 expression was noted in 3 ATLL cases and 1 PTCL-NOS case. The expression of SOX4 was significantly higher in ATLL than in PTCL-NOS (*p* < 0.001).

**Figure 4:**
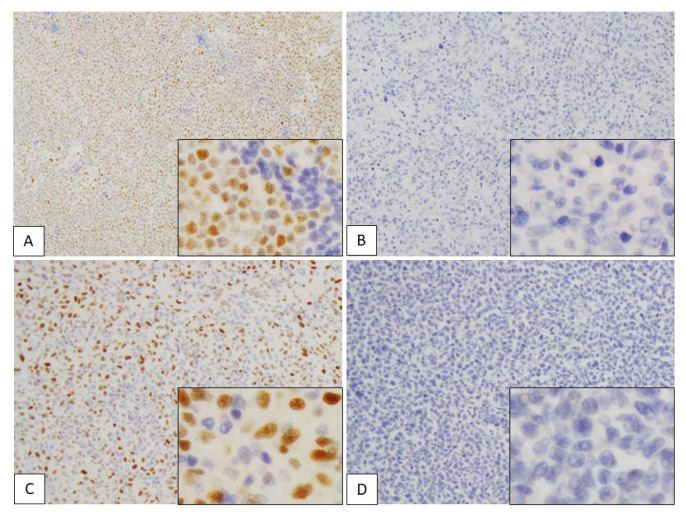
Immunohistochemical assessment of SOX4 in adult T-cell leukemia/lymphoma (ATLL) and peripheral T-cell lymphoma, not otherwise specified (PTCL-NOS). (**A**–**D**) SOX4 expression in the nuclei of lymphoma cells. (**A**) ATLL case showing high expression (positive cells score 3 + stain intensity score 3 = total score 6). (**B**) ATLL case showing no expression (positive cells score 0 + stain intensity score 0 = total score 0). (**C**) PTCL-NOS case showing high expression (positive cells score 3 + stain intensity score 3 = total score 6). (**D**) PTCL-NOS case showing no expression (positive cells score 0 + intensity score 0 = total score 0). Each subpanel shows positive cells from a high-power field of view (×400).

Table 1 and Figure 3 summarize the results of our immunohistochemical analyses in the present study. SOX4 expression in the nucleus of lymphoma cells was considered to be a positive result. In total, 8/11 (73%) patients with ATLL were positive for SOX4, while expression of SOX4 was observed in 2/20 (10%) patients with PTCL-NOS (Figure 4; *p* < 0.001).

In the ATLL group, all of the SOX4-positive cases exhibited high expression (scores of 5–6), while 3 cases showed no SOX4 expression (score 0). On the other hand, 1 of the 2 SOX4-positive cases in the PTCL-NOS group showed high expression of SOX4 (score 5–6), while the other exhibited low expression (score 1–4). The other 18 cases did not show any SOX4 expression (score 0) (*p* < 0.001). The mean total score of the ATLL group was 4.2 (standard deviation (SD): 0.61), while that of the PTCL-NOS group was 0.50 (SD: 0.46) (*p* < 0.001).

### 3.3. p16-Postive Scoring of ATLL and PTCL-NOS Specimens

**Figure 5:**
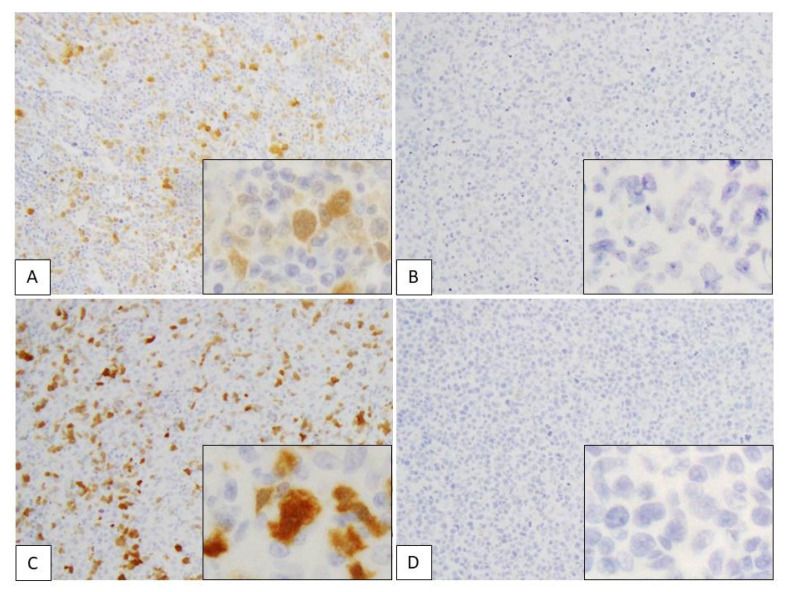
Immunohistochemical assessment of p16 in adult T-cell leukemia/lymphoma (ATLL) and peripheral T-cell lymphoma, not otherwise specified (PTCL-NOS). (**A**–**D**) p16 expression in nuclei and cytoplasm of lymphoma cells. (**A**) ATLL case showing high expression (positive cells score 3 + stain intensity score 3 = total score 6). (**B**) ATLL case showing no expression (positive cells score 0 + stain intensity score 0 = total score 0). (**C**) PTCL-NOS case showing high expression (positive cells score 3 + stain intensity score 3 = total score 6). (**D**) PTCL-NOS case showing no expression (positive cells score 0 + stain intensity score 0 = total score 0). Each subpanel shows positive cells from a high-power field of view (×400).

Positive p16 expression was evident in the nucleus and cytoplasm of lymphoma cells (Figure 5). In total, 4/11 (36%) ATLL cases were positive for p16, while expression of this protein was noted in 3/20 (15%) cases in the PTCL-NOS group (Table 1 and Figure 3; *p* = 0.117). In the ATLL group, 3/4 (75%) of the p16-positive cases had high expression (score 5–6), and 1/4 (25%) exhibited low expression (score 1–4). No p16 expression (score 0) was observed in 7 cases. On the other hand, in the PTCL-NOS group, p16 positivity was noted in 3/20 (15%) cases, including 1/3 (33.3%) with high expression (score 5–6) and 2/3 (66.7%) with low expression (score 1–4). A total of 17/20 cases showed no p16 expression (score 0) (*p* = 0.185). The mean total score of the ATLL group was 1.9 (SD: 0.64), while it was 0.70 (SD: 0.48) in the PTCL-NOS group (*p* = 0.141).

## 4. Discussion

This study used immunohistochemical analysis of SOX4 and p16 expression in patients with ATLL and PTCL-NOS to determine whether these factors may be helpful to discriminate between these diseases. Our results revealed that the SOX4 expression and intensity scores were significantly higher in the ATLL cases than the PTCL-NOS cases. While no statistically significant differences were noted, the p16 expression and intensity scores of the ATLL cases were also higher than those of the PTCL-NOS cases.

In ATLL, the HTLV-1 Tax protein is crucial to the carcinogenic process [20,21,22,23]. Tax is thought to cause carcinogenesis by various mechanisms such as upregulating expression of interleukin (IL)-2, IL-2R-α, and IL-15, which induce the expression of genes involved in T-cell growth and proliferation [24]. However, ATLL cells do not necessarily express Tax at later stages of carcinogenesis [25,26,27] and it is possible that numerous genetic and epigenetic changes may play a role in the multistep carcinogenic process responsible for ATLL. Previous reports have described the occurrence of *p53* mutations and *CDKN2A* (*p16*) deletions in ATLL [28,29,30,31,32]. Inactivation of *p16* has been found in nearly 50% of all human cancers, caused by deletion, methylation, or gene mutations, leading to alterations in protein production [14]. Since the frequency of *p16* deletion is reported to increase as the ATLL stage progresses [28], we expected that patients with ATLL might have lower scores for p16 expression in our immunostaining analyses compared to those with PTCL-NOS. However, no significant difference in p16 expression was observed between the ATLL and PTCL-NOS groups. Indeed, the ATLL group had higher scores for p16-positive cells and intensity, which suggests that ATLL oncogenesis involves a number of factors independent of *p16* gene deletion. Therefore, immunohistochemical staining for p16 might not be as helpful to distinguish between ATLL and PTCL-NOS as we initially hypothesized.

In contrast, the SOX4-positive cells’ scores and staining intensity were significantly higher in the ATLL group than in the PTCL-NOS group. *SOX4* is frequently overexpressed in a variety of solid tumors and is considered to be a potential oncogene. Higuchi et al. reported that *SOX4* is consistently expressed at both mRNA and protein levels in ATLL [11]. *SOX4* may play a role in ATLL carcinogenesis because its expression is induced (via transforming growth factor β (TGF-β)) by the HTLV-1 basic leucine zipper (HBZ) factor. In a previous study [33,34,35,36], *SOX4* was shown to be consistently expressed in primary blood-circulating and skin-infiltrating ATLL cells. Furthermore, the study suggests that SOX4 was involved in ATLL cell growth. In the current study, the SOX4-positive cases comprised 8/11 (73%) ATLL and 2/20 (10%) PTCL-NOS cases. These results suggest that a high SOX4-positive cell score in immunohistochemical staining may be helpful to differentiate between ATLL and PTCL-NOS. Although the oncogenic cascade of ATLL is not yet fully elucidated, it has been suggested that describing the mechanisms of expression of *SOX4* more clearly could reveal its potential as a therapeutic target molecule. While serum anti-HTLV-1 antibody positivity is required for ATLL diagnosis [37], this information is often not available at the time of pathological diagnosis. As noted above, the recommended treatment regimens for ATLL and PTCL-NOS are different, which requires pathologists to differentiate between these two entities despite the difficulties involved. One limitation of the present study was the limited number of cases available for analysis due to the rarity of these diseases. However, despite the small number of cases, our results suggest that SOX4 immunohistochemical staining may provide a valuable tool in addressing the challenge of differential diagnosis for these cancers. Future studies are warranted to confirm the differences in the extent of SOX4 gene expression between ATLL and PTCL-NOS, and to investigate whether there are any correlations between the intensity of SOX4 expression in tumor cells and the clinical prognosis of ATLL patients.

## Figures and Tables

**Table 1:** SOX4 and p16 immunohistochemical staining and scores of patients with adult T-cell leukemia/lymphoma (ATLL) and peripheral T-cell lymphoma, not otherwise specified (PTCL-NOS).

	ATLL (*n* = 11)	PTCL-NOS (*n* = 20)	*p*-Value
	Prevalence (%) or Mean (SD)	Prevalence (%) or Mean (SD)	
**SOX4**			
Positive Cell Score (0–3)	2.0 (0.29)	0.20 (0.21)	<0.001
Stain Intensity (0–3)	2.2 (0.34)	0.30 (0.25)	<0.001
Total Score	4.2 (0.61)	0.50 (0.46)	<0.001
**Extent of Expression** High Low No	8/11 (72.7) 0/11 (0) 3/11 (27.3)	1/20 (5.0) 1/20 (5.0) 18/20 (90.0)	
**p** **16**			
Positive Cell Score (0–3)	0.82 (0.28)	0.25 (0.21)	0.117
Stain Intensity (0–3)	1.1 (0.38)	0.45 (0.28)	0.185
Total Score	1.9 (0.64)	0.70 (0.48)	0.141
**Extent of Expression** High Low No	3/11 (27.3) 1/11 (9.1) 7/11 (63.6)	1/20 (5.0) 2/20 (10.0) 17/20 (85.0)	

## Data Availability

The datasets generated and analyzed during the current study are available from the corresponding author on reasonable request.
